# Water Quality Assessment of Tributaries of Batang Baleh in Sarawak Using Cluster Analysis

**DOI:** 10.1155/2018/8682951

**Published:** 2018-12-02

**Authors:** Teck-Yee Ling, Chen-Lin Soo, Teresa-Lee-Eng Heng, Lee Nyanti, Siong-Fong Sim, Jongkar Grinang, Karen-Suan-Ping Lee, Tonny Ganyai

**Affiliations:** ^1^Faculty of Resource Science and Technology, Universiti Malaysia Sarawak, 94300 Kota Samarahan, Sarawak, Malaysia; ^2^Research and Development Department, Sarawak Energy Berhad, 93050 Kuching, Sarawak, Malaysia

## Abstract

Assessment of river water quality is essential as it provides the knowledge required to make informed decisions. Therefore, water quality was determined at 15 tributary stations located along the Batang Baleh, Sarawak. Results of the study indicate that all tributaries were well-aerated (≈ 7.7 mg/L) with pH (≈ 7.3) and conductivity (≈ 37.3 *μ*S/cm) values falling within acceptable ranges. However, there were tributaries that showed very high turbidity (> 1000 NTU) and suspended solids (> 800 mg/L) which were contributed by the soil erosion from logging activities in the watershed. Tributary stations associated with logging activities also showed significantly higher total phosphorus and organic nitrogen. Cluster analysis demonstrated that water quality at tributary stations along the Batang Baleh exhibited a longitudinal variation from upstream to downstream regions, particularly, dissolved oxygen, five-day biochemical oxygen demand, and nitrite-nitrate nitrogen, which were found higher in upstream region and steadily decreased towards the downstream region. Two stations located at Sg. Serani and Sg. Melatai were distinct from the other stations with the highest concentrations of turbidity, total suspended solids, organic nitrogen, and total phosphorus. Thus, there is an urgent need to reduce the pollutants in the tributaries of Batang Baleh for the health of the sensitive aquatic organisms.

## 1. Introduction

Batang Baleh is one of the main tributaries of the Rajang River (551 km) which is the longest river in Malaysia. These rivers flow through the Kapit Division which is a forested mountainous region. The area is characterized as dipterocarp forest and has been subjected to logging activities for decades. Logging activities have been known to increase suspended solids and nutrients [1–6]. As annual rainfall measured at Kapit is among the highest in Sarawak which exceeds 5000 mm in most years, the impact of logging activities could be more severe in the area due to the surface runoff which contains high suspended solids and nutrients from logging sites [7, 8].

Water quality deterioration has a great influence on the aquatic biota and the ecosystem of a river. The increase in suspended solids limits the light penetration which has major impacts on algae and macrophytes while nutrient enrichment can lead to the depletion of oxygen and subsequently fish kill [9–11]. Exposure to high turbidity and suspended solids impacts fish growth and increases the mortality of fish [12–16]. Hence, water quality monitoring is important in order to evaluate the quality of the river for the health of sensitive aquatic organisms. The baseline data is also useful in management decision for improving and protecting the environment.

Although the nutrient content of water draining forested watersheds is generally lower than domestic and agricultural runoff [17], the potential impacts of the high suspended solids and nutrients from the logging activities to the rivers cannot be overlooked. Deteriorated water quality has been reported at other tributaries of major rivers in Sarawak [8, 18]. Water quality monitoring of those tributaries tends to generate a large set of data. Application of multivariate statistical analysis is useful in analyzing the spatial variation of water quality in the study area. Hence, this study aimed to determine the water quality of the tributaries located along the Batang Baleh which is subjected to logging activities and to assess the spatial variation of the tributary water quality by the integration of cluster analysis.

## 2. Materials and Methods

**Figure 1:**
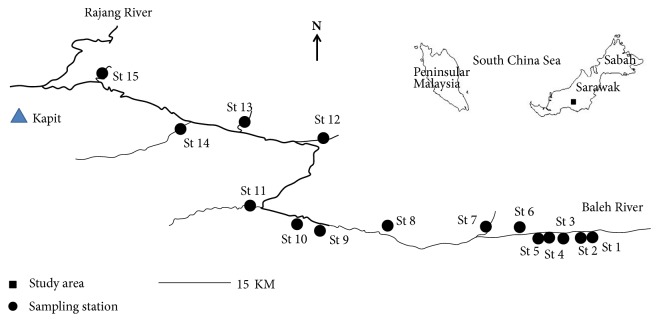
The study area and sampling stations in the present study.

Field samplings were carried out along the Batang Baleh in Sarawak, Malaysia, as indicated in Figure 1. All sampling stations were located at the tributaries of the Batang Baleh. A total of 15 tributary stations were selected along the Batang Baleh from upstream to downstream direction (Table 1). More longhouses were located at the downstream area.

The methods previously described in Ling et al. [19] were used in the present study to obtain data for* in situ* parameters of water temperature, dissolved oxygen (DO), pH, conductivity, and turbidity and* ex situ *parameters of chlorophyll* a* (chl* a*), total suspended solids (TSS), five-day biochemical oxygen demand (BOD_5_), total ammonia nitrogen (TAN), organic nitrogen (Org-N), and total phosphorus (TP). For NO_2_^−^-N + NO_3_^−^-N (nitrite-nitrate nitrogen) analysis, filtration of the sample was conducted using a 0.7 *μ*m retention glass fibre filter (Sartorius Stedim MGF) and it was subsequently acidified to pH < 2. All the samples for* ex situ* analyses were cooled with ice in a cooler box while being transported to the laboratory with the exception of BOD_5_ [20].

All water analyses were performed according to standard procedures [20, 21]. In brief, chl* a* was determined from adequate sample filtered through a 0.7 *μ*m glass fibre filter (Sartorius Stedim MGF) and extracted for 24 h using 90 % (v/v) acetone. TSS was assayed as the difference between the initial and final weights of the 1.0 *μ*m retention glass fibre filter (Sartorius Stedim MGC), after filtration of an adequate sample volume and drying at 105°C. BOD_5_ of the undiluted sample was determined as the difference between the initial and final DO contents after five days of incubation. NO_2_^−^-N + NO_3_^−^-N was determined by the cadmium reduction method followed by the diazotization method (low range) whereas TAN was determined by Nessler's method after the distillation of samples. Org-N was determined by the Macro-Kjeldahl method where ammonia was removed from the water sample before digestion and distillation. Subsequently, ammonia was analyzed by using Nessler's method. TP was determined by the ascorbic acid method after persulfate digestion of samples. A calibration curve was constructed for each chemical analysis. Blank and standard solutions were treated in the same way as the samples.

For each physicochemical parameter, significant difference between the stations was conducted using one-way ANOVA. If there is a significant difference (*p* value ≤ 0.05) among the stations, pairwise comparisons were conducted using Tukey's test. Pearson's correlation analysis was performed to determine the relationship among all the parameters. Cluster analysis (CA) was used to identify the grouping of the stations by using the physicochemical parameters. *Z*-score standardization of the variables and Ward's method using Euclidean distances as a measure of similarity was used. The cluster was considered statistically significant at a linkage distance of < 60% and the number of clusters was decided by the practicality of the outputs [22]. All the statistical analyses were carried out by using the Statistical Software for Social Sciences (SPSS Version 22, SPSS Inc., 1995)

## 3. Results and Discussion

*Tributary Water Quality*. Tributary stations were relatively shallow in the study area, ranging from 0.15 ± 0.09 m to 3.10 ± 0.00 m (Table 2). The depths of those stations were significantly different (*p* value ≤ 0.05) and they demonstrated an increasing trend from upstream to downstream regions. Temperature values of the forest streams in the present study ranged from 24.2 ± 0.0°C to 26.6 ± 0.0°C and they exhibited significant difference (*p* value ≤ 0.05) between stations. Temperature was found related to the sampling time as the water was cooler in the morning whereas the temperature of streams increased significantly (*p* value ≤ 0.05) in the afternoon. Similar result was demonstrated by Ling et al. [8] where the authors attributed the large variation in temperature of the forest stream on the same day to the canopy removal in the study area.

There was no sign of acidification of the forest streams as indicated by pH ≥ 7 and all streams were well-aerated with DO ≥ 6.8 mg/L. However, DO values were observed to be significantly lower (*p* value ≤ 0.05) at the downstream region of the Batang Baleh particularly at stations 11, 13, 14, and 15. Those lower DO values were attributed to the higher organic matter as the decomposition process consumed the DO rapidly. The higher organic matter was due to the organic waste, grey water, and partially treated black water discharged from the residents of the logging camps and longhouses and organic materials associated with logging activities located upstream of those sampling stations [2]. The DO value was also found significantly and positively correlated (*p* value ≤ 0.05) with BOD_5_ and Org-N in the present study. Ling et al. [18] also attributed the positive correlation between DO and pollutants to the rapid aeration and high surface runoff in a fast flowing river. The pH value of tributary stations fluctuated along the Batang Baleh with the lowest and the highest pH values observed at station 5 and station 6, respectively. Conductivity value of the forest streams ranged from 19.3 ± 0.2 *μ*S/cm to 54.0 ± 0.0 *μ*S/cm where significantly lower (*p* value ≤ 0.05) conductivity values were found at tributary stations that were located in the middle part of the Batang Baleh. The pH, DO, and conductivity values at all tributary stations were classified as Class I according to the National Water Quality Standard (NWQS) for Malaysia [23].

In the present study, the turbidity and TSS values ranged from 12.6 ± 0.0 NTU to 1159.5 ± 0.0 NTU and 12.7 ± 2.5 mg/L to 888.3 ± 97.5 mg/L, respectively. Turbidity was classified as Class II at most of the stations except for the five stations (7, 9, 11, 13, and 15) where the guideline value of 50 NTU for the health of sensitive aquatic organisms was exceeded. Similarly, TSS was classified as Class I or II at most of the stations except stations 11 and 12 (Class III) and stations 7 and 9 (Class V). The high turbidity and TSS values observed at stations 7, 9, 11, and 12 were due to soil erosion from logging activities upstream resulting in the sediment influx through surface runoff. It has been reported that in Malaysia logging or ground clearance increased river sediment yields by two to fifty times [3]. Additionally, results of a recent study conducted in Sarawak showed that logging and associated activities induced the formation of soil erosion hotspots which remained for several years and that even though the exposed barren land resulting from logging activities only covered over 4% of the study area, they contributed more than 28% of the total soil loss [6]. The eroded soil ends up in the receiving stream increasing the turbidity and TSS. Similar observations of a significant increase in suspended solids after clear-cut timber harvesting were reported [2, 4]. The extremely high values of turbidity and TSS observed at the tributary stations of the present study were substantially higher than those recorded at the tributaries of the Baram River where the highest turbidity and TSS were about 468 NTU and 320 mg/L, respectively, and they were also attributable to logging activities [18, 19].

Chl* a* concentration ranged from 0.02 ± 0.00 mg/m^3^ to 1.36 ± 0.21 mg/m^3^ at tributary stations of the Batang Baleh (Table 3). Significantly lower (*p* value ≤ 0.05) chl* a* concentrations were observed at tributary stations that were located in upper part of the Batang Baleh than those in lower part of the river. The highest value of chl* a* was observed at station 9 followed by station 15 which were significantly higher (*p* value ≤ 0.05) than those at the other stations. The high chl* a* at stations 9 and 15 were due to the available nutrients from settlements along the rivers as station 9 recorded the second highest in TP and both stations exceeded Class II limit of 0.2 mg/L in TP. Significant and positive correlation (Table 4, *p* value ≤ 0.05) between turbidity, TSS, and chl* a *were observed in the present study indicating that phytoplankton contributed to the turbidity and suspended solids readings. In contrast to turbidity and TSS, chl* a* concentrations in the present study were substantially lower than those in the tributaries of the Baram River located at the same region [18, 19]. The highest chl* a* concentration at the tributary station of the Baram River was approximately 26 mg/m^3^ which is extremely high compared to the present study (< 2 mg/m^3^).

The BOD_5_ concentrations steadily decreased from upstream to downstream regions, ranging from 0.22 ± 0.19 mg/L to 3.28 ± 0.50 mg/L. Significantly higher (*p* value ≤ 0.05) BOD_5_ concentrations were observed at tributary stations that were located at the upper part of the Batang Baleh than those at the lower part of the river. BOD_5_ concentrations at stations 1-3 were the highest among the stations because of the high organic matter from plant debris of past logging activities which accumulated in those streams. BOD loads due to logging debris were also cited as a factor for the significant decrease in DO after clear-cut timber harvesting [2]. DOC (dissolved organic carbon) was also reported to increase strongly in concentration after forest operations in boreal first-order streams [5]. The high BOD_5_ also explains the significantly depressed DO observed in those streams even though the streams were fast flowing and well-aerated mountain streams. Similar observation of higher BOD_5_ concentrations at the tributaries located at the upper part of the Baram River was also reported [18]. Tributary stations at the upper part of the Batang Baleh were mostly classified as Class II and/or III whereas tributary stations at downstream region were classified as Class I and/or II. BOD_5_ was positively correlated with DO and NO_2_^−^-N + NO_3_^−^-N (Table 4, *p* value ≤ 0.05) revealing the active decomposition process of the organic matter where oxygen is being consumed by the bacteria to decompose the organic matter. The chl* a*, TSS, and BOD_5_ concentrations at station 11 (Sg. Mengiong) and station 14 (Sg. Gaat) in the present study were relatively lower than those at the upper part of the streams [8]. This reveals that the sources of pollutant are most probably located at the upstream of the watershed and dilution occurs along the streams.

The NO_2_^−^-N + NO_3_^−^-N concentration was relatively low in the study area, ranging from below detection limit to 0.080 ± 0.020 mg/L. Similar to BOD_5_, the NO_2_^−^-N + NO_3_^−^-N concentration was also significantly higher (*p* value ≤ 0.05) at tributary stations that were located at upper part of the Batang Baleh than those at the lower part of the river. The higher NO_2_^−^-N + NO_3_^−^-N is most likely due to increased leaching from the soil after precipitation as mineralization of organic matter was enhanced after timber harvesting and reduced uptake of nutrients by plants [1] and also the conversion of TAN to NO_2_^−^-N + NO_3_^−^-N as the concentrations corresponded to lower TAN in particular stations 1-4 as the streams were fast flowing well-aerated mountain after rainfall events. The TAN concentration at most stations were classified as Class II except stations 2, 3, and 4 (≈ 0.07 mg/L) which were classified as Class I and station 15 (0.37 ± 0.03 mg/L) which was classified as Class III. TAN concentration at station 15 was also significantly (*p* value ≤ 0.05) higher than that at the other stations due to the organic waste including domestic animals from the households in the longhouses upstream of the station. Org-N concentration was found high at tributary stations that were located in the middle part of the Batang Baleh. The lowest and the highest concentrations of Org-N were observed at station 13 (0.10 ± 0.01 mg/L) and station 7 (0.53 ± 0.04 mg/L), respectively. TP concentration fluctuated at the tributary stations along the Batang Baleh where around a half of the sampling stations complied with the guideline value of 0.2 mg/L but the other half exceeded the guideline value. The highest concentration of TP was observed at station 7 (0.85 ± 0.06 mg/L), followed by station 9 (0.50 ± 0.01 mg/L) which were significantly higher (*p* value ≤ 0.05) than the other stations. Similar to turbidity and TSS, nutrients such as Org-N and TP were also the highest among the stations at stations 7 and 9 due to the decaying plants debris from logging activities, the wash down of particulate organic matter following the disturbance of the forest floor during timber harvesting activities, and a reduction in the plant uptake of nutrients in the watershed [2]. A study on the impact of clear-cut logging also reported the observation of an increase in the concentration of TN and TP at the outlet of the catchments [4]. TP was significantly and positively correlated with turbidity, TSS, and Org-N (Table 4, *p* value ≤ 0.05) in the present study indicating that phosphorus might be attached with suspended solids and brought together into forest streams [10]. Despite the high turbidity and suspended solids, the nutrients content of the tributary stations of the Baleh River was relatively lower than the Baram River with mean values of 0.33 mg/L of TP, 0.54 mg/L of TAN, and 0.99 mg/L of Org-N [18]. The lower nutrients content in tributary stations of the Baleh River in the present study also explains the lower chl* a *concentration than the Baram River.

**Figure 2:**
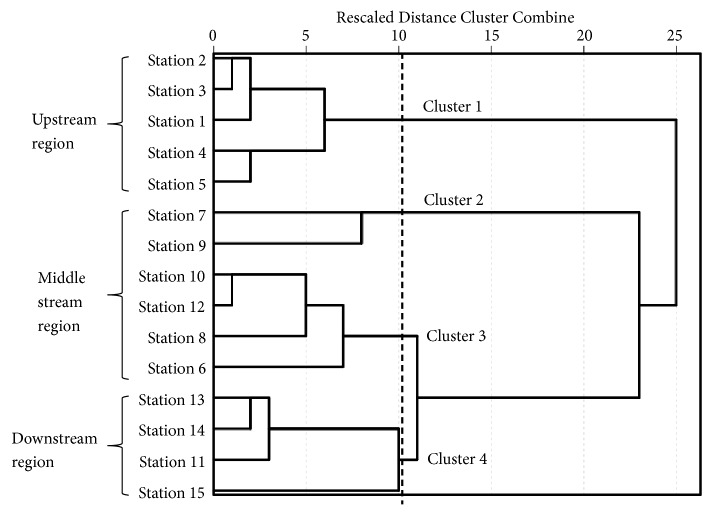
Dendrogram of the cluster analysis.

Cluster analysis (CA) was applied to detect similarities among the tributary stations of the Batang Baleh. It demonstrated that water quality at tributary stations along the Batang Baleh exhibited a longitudinal variation from upstream to downstream areas. The dendrogram shows that the 15 tributary stations can be grouped into four clusters according to the proximity of those stations (Figure 2). Generally, tributary stations along the Batang Baleh are grouped according to upstream, middle, and downstream regions. Cluster 1 consists of stations located in upstream region, clusters 2 and 3 are mostly stations that were located in the middle stream region, and cluster 4 consists of stations that were located in downstream region. Tributary stations that were located in upstream region (cluster 1) are mostly higher in BOD_5_ and NO_2_^−^-N + NO_3_^−^-N than tributary stations in downstream region (cluster 4). Stations 7 and 9 are grouped together in cluster 2 where these two stations were the most polluted stations with the highest concentrations of turbidity, TSS, and TP. Both of the stations also contained high Org-N, but were low in conductivity and NO_2_^−^-N + NO_3_^−^-N. Similarly, Merit River which is one of the tributaries of the Batang Rajang was also identified as more polluted than the other tributaries using cluster analysis [24].

## 4. Conclusions

The tributary water quality of the Batang Baleh was determined and the results show that pH, DO, and conductivity were classified as Class I and/or II according to the NWQS for Malaysia. Turbidity value was high where all stations are classified as Class II and/or exceeded Class II. Two tributaries showed extremely high TSS thus classified as Class V. High turbidity and TSS values at those stations are attributable to erosion as a result of logging activities. Other than that, the TSS, BOD_5_, and TAN values fluctuated among the tributaries, ranging from Class I to Class III. Half of the tributary stations along the Batang Baleh contained high TP value that exceeded the guideline value. Water quality at tributary stations along the Batang Baleh exhibited a longitudinal variation from upstream to downstream regions; in particular, those in the upstream region were mostly higher in DO, BOD_5_, and NO_2_^−^-N + NO_3_^−^-N than tributary stations in downstream region. Low DO and high TAN value in downstream region particularly station 15 could be attributed to the domestic wastewater discharged from residential areas such as longhouses.

## Figures and Tables

**Table 1:** The details of the sampling regime and sampling location in the present study.

Station	GPS Coordinate	Date	Time	Remark
St 1: Sg Selentang	N01°34′05.6′′ E114°21′14.0′′	21/11/2015	3:00 pm	Raining
St 2: Sg. Penganen	N01°34′02.0′′ E114°20′47.2′′	21/11/2015	2:25 pm	Raining
St 3: Sg. Irak	N01°33′55.2′′ E114°18′38.9′′	21/11/2015	1:35 pm	Raining
St 4: Sg. Tor	N01°34′04.3′′ E114°16′45.8′′	21/11/2015	11:09 am	Raining
St 5: Sg. Kian	N01°34′01.1′′ E114°15′47.2′′	21/11/2015	10:30 am	Raining
St 6: Sg. Kupet	N01°33′54.2′′ E114°12′53.0′′	5/4/2015	12:50 pm	Sunny
St 7: Sg. Serani	N01°34′16.5′′ E114°08′40.7′′	5/4/2015	11:33 am	Shaded and sunny
St 8: Sg. Laie	N01°34′06.3′′ E113°55′38.3′′	4/4/2015	4:37 pm	Shaded and sunny
St 9: Sg. Melatai	N01°35′21.9′′ E113°47′44.7′′	4/4/2015	1:31 pm	Shaded and sunny
St 10: Sg. Entuloh	N01°36′12.2′′ E113°44′40.6′′	4/4/2015	11:52 am	Shaded and sunny
St 11: Sg. Mengiong	N01°37′55.4′′ E113°38′05.0′′	12/2/2015	2:20 pm	Sunny
St 12: Sg. Putai	N01°48′45.4′′ E113°46′14.2′′	12/2/2015	4:25 pm	Raining
St 13: Sg. Merirai	N01°51′36.1′′ E113°34′39.1′′	13/2/2015	8:55 am	Sunny
St 14: Sg. Gaat	N01°52′30.5′′ E113°26′08.6′′	13/2/2015	10:25 am	Partially cloudy
St 15: Sg. Mujong	N02°01′39.5′′ E113°10′54.9′′	13/2/2015	1:00 pm	Raining

**Table 2:** Summary of in situ parameter at the tributary station of the Batang Baleh.

Station	Depth, m		Temperature, °C		pH			DO, mg/L			Conductivity, *μ*S/cm			Turbidity, NTU		
	Mean	SD	Mean	SD	Mean	SD	Class	Mean	SD	Class	Mean	SD	Class	Mean	SD	Class
St 1	0.24^a^	0.05	25.1^de^	0.0	7.5^g^	0.0	I	7.79^cd^	0.00	I	39.0^g^	0.1	I	47.0^bc^	0.0	II
St 2	0.25^a^	0.14	25.0^d^	0.0	7.4^f^	0.0	I	7.92^ef^	0.00	I	49.0^l^	0.0	I	47.8^bc^	0.3	II
St 3	0.16^a^	0.06	25.2^ef^	0.0	7.2^bc^	0.0	I	7.91^e^	0.00	I	48.0^k^	0.0	I	21.0^a^	0.1	II
St 4	0.15^a^	0.09	24.5^b^	0.0	7.1^b^	0.0	I	7.95^ef^	0.00	I	43.0^i^	0.0	I	18.2^a^	0.1	II
St 5	0.15^a^	0.04	24.2^a^	0.0	7.0^a^	0.0	I	8.04^fg^	0.01	I	40.0^h^	0.0	I	15.5^a^	0.1	II
St 6	0.60^b^	0.00	24.8^c^	0.0	7.9^i^	0.0	I	8.14^g^	0.00	I	54.0^m^	0.0	I	38.7^b^	0.2	II
St 7	1.27^c^	0.06	24.7^c^	0.0	7.3^e^	0.0	I	8.10^g^	0.00	I	23.0^c^	0.0	I	477.0^g^	10.8	> II
St 8	1.90^d^	0.00	26.6^i^	0.0	7.4^f^	0.0	I	7.68^c^	0.00	I	30.0^d^	0.0	I	12.6^a^	0.0	II
St 9	1.77^d^	0.06	25.4^g^	0.0	7.2^cd^	0.0	I	8.00^efg^	0.00	I	22.0^b^	0.0	I	1159.5^h^	0.0	> II
St 10	0.70^b^	0.00	25.3^fg^	0.0	7.5^g^	0.0	I	7.94^de^	0.00	I	34.0^e^	0.0	I	22.2^a^	0.0	II
St 11	2.50^f^	0.00	24.7^c^	0.1	7.2^d^	0.0	I	7.13^b^	0.06	I	19.3^a^	0.2	I	122.4^e^	2.4	> II
St 12	1.30^c^	0.00	25.0^d^	0.1	7.3^e^	0.0	I	7.77^c^	0.06	I	33.9^e^	0.1	I	48.8^c^	0.0	II
St 13	3.10^h^	0.00	25.0^d^	0.1	7.6^h^	0.0	I	7.17^b^	0.06	I	45.8^j^	0.1	I	106.5^d^	2.7	> II
St 14	2.20^e^	0.00	25.1^de^	0.1	7.2^cd^	0.0	I	6.83^a^	0.06	II	40.1^h^	0.1	I	49.5^c^	3.6	II
St 15	2.70^g^	0.00	25.7^h^	0.1	7.2^cd^	0.0	I	6.90^a^	0.10	II	38.5^f^	0.1	I	185.7^f^	3.6	> II

Same letters indicate no significant difference at *p* value > 0.05.

**Table 3:** Summary of water quality at the tributary station of the Batang Baleh.

Station	Chl *a*, mg/m^3^		TSS, mg/L			BOD_5_, mg/L			TAN, mg/L			NO_2_^−^-N + NO_3_^−^-N, mg/L		Org-N, mg/L		TP, mg/L		
	Mean	SD	Mean	SD	Class	Mean	SD	Class	Mean	SD	Class	Mean	SD	Mean	SD	Mean	SD	Class
St 1	0.02^a^	0.00	47.0^ab^	5.6	II	3.28^e^	0.50	III	0.10^ab^	0.03	II	0.073^e^	0.006	0.28^bc^	0.03	0.40^d^	0.03	> II
St 2	0.10^ab^	0.02	42.3^ab^	5.0	II	3.02^e^	0.26	III	0.08^a^	0.02	I	0.050^d^	0.010	0.26^bc^	0.00	0.08^ab^	0.00	I
St 3	0.11^abc^	0.01	17.3^a^	1.5	I	3.26^e^	0.44	III	0.07^a^	0.01	I	0.027^c^	0.006	0.37^cde^	0.03	0.34^cd^	0.01	> II
St 4	0.23^abcd^	0.01	17.3^a^	2.9	I	2.55^de^	0.11	II	0.07^a^	0.01	I	0.080^e^	0.020	0.36^cde^	0.06	0.28^c^	0.03	> II
St 5	0.11^ab^	0.01	17.7^a^	2.1	I	2.31^de^	0.04	II	0.21^e^	0.04	II	0.060^de^	0.010	0.44^ef^	0.00	0.14^b^	0.01	I
St 6	0.35^d^	0.07	33.0^ab^	2.0	II	1.98^cd^	0.37	II	0.14^bcd^	0.01	II	0.003^ab^	0.006	0.30^bcd^	0.02	0.03^a^	0.01	I
St 7	0.39^d^	0.04	486.7^c^	8.7	V	2.98^de^	0.08	II	0.14^bcd^	0.01	II	0.013^abc^	0.006	0.53^f^	0.04	0.85^f^	0.06	> II
St 8	0.26^bcd^	0.04	14.3^a^	1.5	I	0.80^ab^	0.14	I	0.16^bcd^	0.02	II	0.010^abc^	0.000	0.43^def^	0.11	0.03^b^	0.00	I
St 9	1.36^f^	0.21	888.3^d^	97.5	V	0.81^ab^	0.50	I	0.17^cde^	0.01	II	0.010^abc^	0.000	0.45^ef^	0.03	0.50^e^	0.01	> II
St 10	0.32^cd^	0.02	20.0^a^	1.7	I	0.91^ab^	0.42	I	0.14^bcd^	0.01	II	0.020^abc^	0.000	0.37^cde^	0.06	0.32^cd^	0.10	> II
St 11	0.23^abcd^	0.05	104.2^b^	3.8	III	1.20^abc^	0.27	II	0.15^bcd^	0.03	II	0.000^a^	0.000	0.12^a^	0.01	0.05^ab^	0.00	I
St 12	0.19^abcd^	0.01	57.7^ab^	4.0	III	1.24^bc^	0.40	II	0.18^de^	0.01	II	0.023^bc^	0.006	0.20^ab^	0.04	0.02^a^	0.00	I
St 13	0.28^bcd^	0.04	26.7^a^	2.9	II	0.22^a^	0.19	I	0.13^abcd^	0.00	II	0.000^a^	0.000	0.10^a^	0.01	0.03^a^	0.00	I
St 14	0.24^bcd^	0.04	12.7^a^	2.5	I	0.74^ab^	0.27	I	0.12^abc^	0.01	II	0.010^abc^	0.000	0.21^ab^	0.01	0.02^a^	0.00	I
St 15	0.71^e^	0.12	39.6^ab^	1.7	II	0.95^ab^	0.47	I	0.37^f^	0.03	III	0.017^abc^	0.006	0.39^cde^	0.07	0.25^c^	0.01	> II

Same letters indicate no significant difference at *p* value > 0.05.

**Table 4:** Correlation matrix of physicochemical parameters collected from the tributary stations along the Batang Baleh.

	Temperature	pH	DO	Conductivity	Turbidity	Chl *a*	TSS	BOD_5_	TAN	NO_2_^−^-N + NO_3_^−^-N	Org-N	TP
Temperature	1.000											
pH	0.174	1.000										
DO	-0.244	0.156	1.000									
Conductivity	-0.174	0.386	0.090	1.000								
Turbidity	0.093	-0.175	0.166	-0.556^*∗*^	1.000							
Chl *a*	0.280	-0.105	-0.018	-0.430	0.893^*∗*^	1.000						
TSS	0.030	-0.168	0.279	-0.599^*∗*^	0.982^*∗*^	0.814^*∗*^	1.000					
BOD_5_	-0.410	-0.090	0.579^*∗*^	0.292	-0.175	-0.428	-0.071	1.000				
TAN	0.262	-0.191	-0.391	-0.237	0.168	0.434	0.070	-0.433	1.000			
NO_2_^−^-N + NO_3_^−^-N	-0.364	-0.325	0.382	0.302	-0.274	-0.389	-0.241	0.658^*∗*^	-0.277	1.000		
Org-N	0.175	-0.276	0.551^*∗*^	-0.224	0.386	0.360	0.435	0.322	0.210	0.193	1.000	
TP	-0.102	-0.192	0.416	-0.402	0.563^*∗*^	0.351	0.638^*∗*^	0.436	-0.040	0.146	0.678^*∗*^	1.000

^*∗*^indicates significant correlation at *p* value ≤ 0.05

## Data Availability

The data used to support the findings of this study are available from the corresponding author upon request.
